# The impact of bronchoalveolar lavage fluid metagenomics next-generation sequencing on the diagnosis and management of patients with suspected pulmonary infection

**DOI:** 10.3389/fcimb.2025.1521641

**Published:** 2025-06-23

**Authors:** Mei Zhou, Shengwen Sun, Long Chen, Huan Xu, Lanlan Liu, Jiaxi Lv, Jianchu Zhang, Xianzhi Xiong

**Affiliations:** ^1^ Department of Respiratory and Critical Care Medicine, NHC Key Laboratory of Pulmonary Diseases, Union Hospital, Tongji Medical College, Huazhong University of Science and Technology, Wuhan, Hubei, China; ^2^ Department of Critical Care Medicine, Union Hospital, Tongji Medical College, Huazhong University of Science and Technology, Wuhan, Hubei, China; ^3^ Department of Scientific Affairs, Vision Medicals for Infectious Diseases, Guangzhou, Guangdong, China

**Keywords:** BALF, mNGS, pulmonary infection, diagnosis, management

## Abstract

**Objectives:**

This study aimed to enhance the comprehension of the practical utility of bronchoalveolar lavage fluid (BALF) metagenomic next-generation sequencing (mNGS) in the clinical management of patients with suspected pneumonia.

**Methods:**

We retrospectively analyzed 296 individuals who underwent BALF mNGS and conventional microbial tests (CMTs) for suspected pneumonia. We compared the clinical characteristics between patients with pulmonary infection (PI) and those without pulmonary infection (NPI). The detection rate of mNGS and CMTs in different groups of patients were compared. The Sankey diagram was used to present the results of the influence of mNGS on diagnosis and treatment.

**Results:**

Comparison between PI and NPI showed that individuals with fever, concurrent malignant tumors, consolidation or ground-glass opacity on chest CT(Computed tomography) images, and elevated inflammatory markers on blood tests were more likely to develop lung infections. Analysis of the rate of positive detection between CMTs and mNGS in various subgroups revealed that mNGS had a significantly higher positive detection rate in patients with pulmonary infections (87.95% vs. 71.06%, p<0.001), in immunocompetent patients (86.91% vs. 68.08%, p<0.001), and in patients with malignant tumors (92.31% vs. 69.23%, p=0.035). Furthermore, mNGS helped initiate appropriate antibiotic treatment and confirmed the effectiveness of empirical treatment. Compared to immunocompetent patients, BALF mNGS in immunocompromised individuals with suspected lung infections yielded higher rates of accurate diagnosis (62.86% vs. 42.79%, p = 0.027) and more effective treatment (71.43% vs. 58.56%, p = 0.148).

**Conclusions:**

BALF mNGS identified a greater variety of pathogens than CMTs. Immunocompromised patients with suspected pneumonia may benefit more from BALF mNGS.

## Introduction

Infectious disease remains to be a global health concern. Despite significant improvements in microbiological testing techniques and medical treatment, the mortality rate remains high (Bloom and Cadarette, 2019). Rapid and accurate etiological diagnosis of pulmonary infection is the fundamental way to control infection, reduce mortality, and prevent the development of drug-resistant bacteria. At present, the primary approach for identifying the etiological pathogens of pulmonary infectious disease is microbial culture. Although it is effective in addressing certain clinical issues, conventional microbiological tests still suffer from some limitations, such as low positive rate, insufficient reliability, and long turnaround time (Li et al., 2019; Sin et al., 2014). In recent years, several rapid diagnostic techniques for identifying the causes of infectious diseases have been applied in healthcare settings. These approaches include antigen/antibody assays and polymerase chain reaction-based nucleic acid detection of specific pathogens (Subramony et al., 2016). However, these tests are typically restricted to healthcare professionals who anticipate potential disease-causing agents, and their outcomes are frequently influenced by thresholds (Dimech, 2021).

Metagenomic next-generation sequencing (mNGS) based on high-throughput sequencing technology has emerged as a solution. Initially, because of its high price and complex operating procedures, mNGS was only used in some scientific research fields (Chiu, 2013; Lloyd-Price et al., 2016). With the display of its superior performance and the reduction of detection cost (van Nimwegen et al., 2016), next-generation sequencing has quickly entered clinical practice, and its application in tumor diagnosis and individualized therapy (Coombs et al., 2018; Tsoulos et al., 2017) and prenatal diagnosis (Xu and Shi, 2014) have been reported. The first application of mNGS in the diagnosis of infectious diseases was reported in the New England Journal in 2014, in which mNGS was used to diagnose an infectious 14-year-old patient with severe combined immunodeficiency syndrome (Wilson et al., 2014). Since then, an increasing number of case reports and clinical studies have pointed out the value of mNGS in the diagnosis of infectious diseases, and research is no longer limited to central nervous system infections (Guan et al., 2016; Yao et al., 2016). Studies on the osteoarticular system (Ruppe et al., 2017), the respiratory system (Leo et al., 2017; Ruppe et al., 2016), and other systems have also been reported.

As a new detection method, mNGS has obvious advantages compared with traditional pathogenic detection methods: (1) It can detect all pathogenic microorganisms simultaneously and quickly; (2) It can quickly detect pathogens that are time-consuming or difficult in traditional pathogenic culture, such as Mycobacterium tuberculosis; (3) Diagnosis of rare and emerging pathogens. At present, there are several challenges in the clinical application of mNGS for diagnosing infectious diseases, such as the absence of standardized protocols, the complexity of interpreting test results, and relatively high expenses (Han et al., 2019). Moreover, it should be noted that the respiratory system is not a sterile environment, which adds complexity to the interpretation of the data. Numerous clinical studies have been published on the utility of mNGS in diagnosing pulmonary infections. However, most of these studies had small sample sizes and were limited to specific populations or pathogens (Lin et al., 2023; Shen et al., 2023; Sun et al., 2022; Wang et al., 2023). BALF mNGS has been used in our department for many years to assist in the diagnosis of pathogens of pulmonary infection. In actual clinical work, we have observed that BALF mNGS has indeed provided great help for the diagnosis and treatment of most patients, but we are not clear about which patients are more suitable for this examination or benefit more. There is a scarcity of studies on the direct impact of BALF mNGS on the diagnosis and treatment of patients with suspected pulmonary infections, including both mild and severe cases. This study was conducted to gain a deeper understanding of the practical application of mNGS in clinical practice.

## Methods

### Study design and patient population

This retrospective study recruited 317 patients from the Department of Respiratory and Critical Care Medicine, between May 2020 and August 2021. This study was conducted in accordance with the principles of the Declaration of Helsinki and approved by the Ethics Committee of Tongji Medical College of Huazhong University of Science and Technology (Grant No. IORG0003571). Given the retrospective nature of the study and the use of anonymized data, the need for written informed consent was waived by the Ethics Committee. All patient information was de-identified prior to analysis to protect patient confidentiality. The inclusion criteria were (1) age≥14 years, (2) suspected pneumonia, and (3) mNGS results. The exclusion criteria were as follows: (1) sample failure to pass the quality control of mNGS, or (2) incomplete clinical data. Based on our inclusion and exclusion criteria, 296 participants were included in the study, while 21 were excluded due to incomplete clinical data caused by random errors in retrieving hospitalization information, such as untraceable or incorrect hospitalization numbers in mNGS reports. No systematic bias related to clinical characteristics (e.g., disease severity or follow-up status) was identified in these exclusions.

In this study, suspected cases of pneumonia met both criteria: (1) new-onset symptoms, such as fever, cough, expectoration, or dyspnea, and (2) new-onset abnormal chest imagine features (Lin et al., 2022). Pulmonary infection was diagnosed using a comprehensive reference standard that included all microbiological tests and clinical adjudication. The reference was based on the diagnostic criteria for community-acquired pneumonia (CAP) (Metlay et al., 2019) and hospital-acquired pneumonia (HAP) (Kalil et al., 2016).

**Figure 1:**
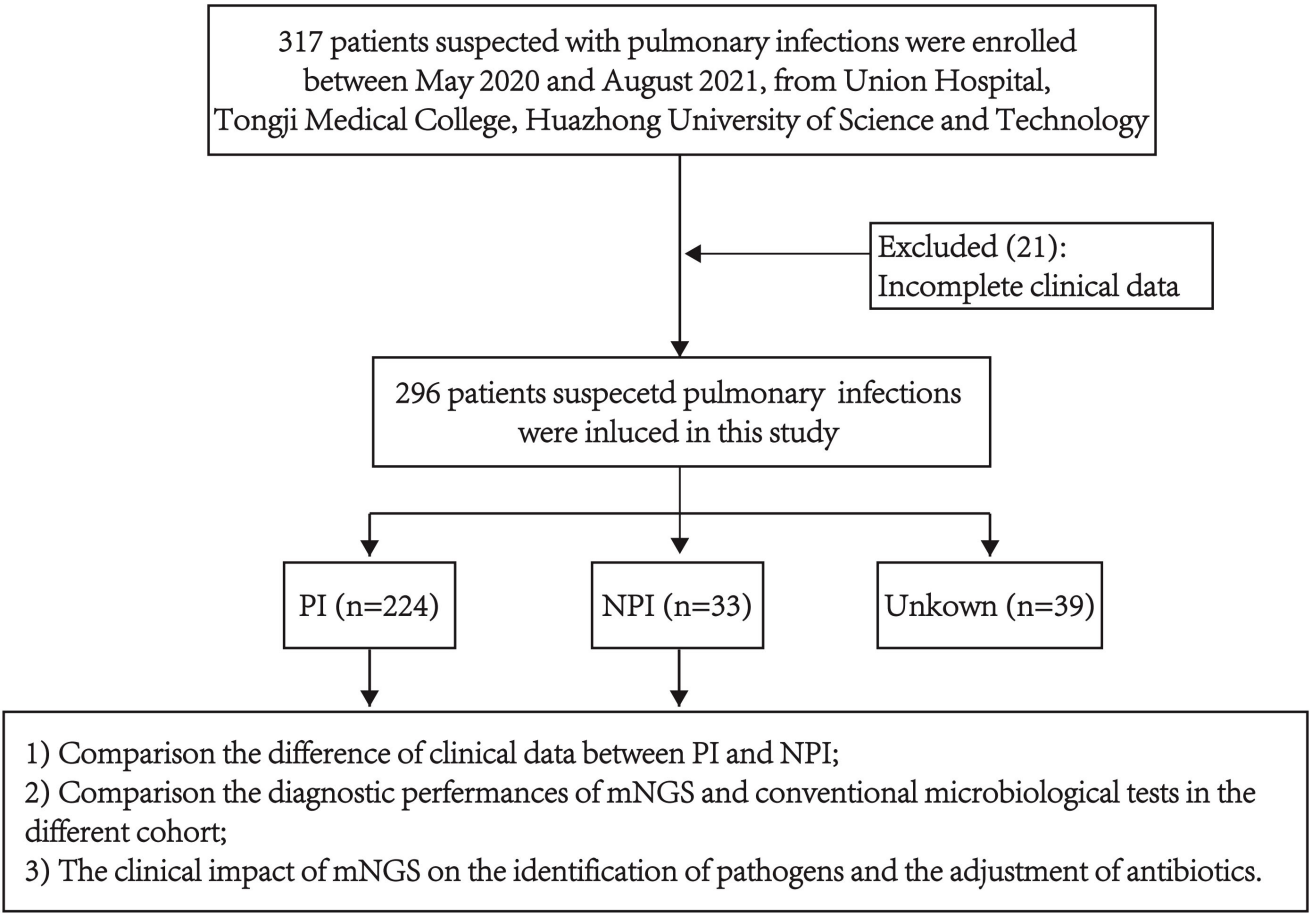
The flowchart of the patients.

We retrospectively analyzed 296 individuals who underwent BALF mNGS and conventional microbial tests (CMTs) for suspected pneumonia. We compared the clinical characteristics between patients with pulmonary infection (PI) and those without pulmonary infection (NPI). Then the detection rate of mNGS and CMTs in different groups of patients were compared (**Figure 1**). In this part, patients were considered immunocompromised if they met any of the following criteria (Ramirez et al., 2020): (1) primary immune deficiency diseases; (2) active malignancy or malignancy within 1 year of CAP, excluding localized skin cancers or early-stage cancers; (3) receiving cancer chemotherapy; (4) HIV infection with a CD4 T-lymphocyte count < 200 cells/μL or a percentage < 14%; (5) solid organ transplantation; (6) hematopoietic stem cell transplantation; (7) receiving corticosteroid therapy with a dose ≥20 mg prednisone or equivalent daily for ≥14 days, or a cumulative dose >600 mg of prednisone; (8) receiving biological immune modulators; (9) receiving disease-modifying antirheumatic drugs or other immunosuppressive drugs. The clinical impact of mNGS was assessed according to the **Supplementary Table 1** and **Supplementary Table 2** (Xu C. et al., 2023).

Taking clinical composite diagnosis as the reference standard, we use manual case-by-case analysis to analyze the influence of mNGS on diagnosis and treatment. The Sankey diagram was used to present the results of data classification and statistics.

### Conventional microbiological tests

In this study, clinicians make preliminary judgments and make necessary CMTs according to the condition and clinical manifestations. Clinical specimens, such as sputum, BALF, blood, pleural effusion, tissue, and bone marrow, were collected. The sputum, BALF, and blood can be used for smear examinations, such as acid-fasting staining (*Mycobacterium. Spp*). An indirect fluorescence immunoassay was used to detect five pathogenic IgM antibodies in the blood, including adenovirus (ADV), Respiratory Syncytial Virus (RSV), *Chlamydia pneumonia* (CP), *Mycoplasma pneumonia* (MP), and Coxsackievirus B5. The blood T-SPOT test, BLAF X-pert test, and acid-fasting staining were performed to detect *Mycobacterium tuberculosis* (MTB). Blood 1, 3-β-D glucan assay (BGD) was performed to detect fungal infection, and blood and BALF galactomannan were used to detect *Aspergillus.* spp infection.

### mNGS of BALF

Specimens such as bronchoalveolar lavage fluid (BALF) must first undergo wall breaking. One gram of glass beads (a diameter of 0.5 mm was added to the wall-breaking tube, followed by 0.6 ml specimen, which was oscillated at a high speed of 2800–3200 rpm for 30 min. and then 300μl was taken for nucleic acid extraction according to the instructions of the Tiangen trace sample genome extraction kit (DP316). 500ng of extracted DNA was taken, and procedures such as interruption, terminal repair, joint addition, amplification, database construction, and sequencing were performed according to standard procedures and sequenced on an Illumina NextSeq 550 sequencer using a 75-cycle single-end sequencing strategy (Huang et al., 2024). The sequence number of the respiratory tract specimens must be greater than 5M. At the same time, internal reference, negative and positive controls were set. The resulting data were stripped of sequences of low quality and too short in length (less than 35bp) to obtain high-quality sequences, which were then compared with the human reference genome (H19) using Burrows-Wheeler Alignment software. After removing human sequences, the sequences were compared with four databases of bacteria, fungi, viruses, and parasites respectively to obtain a sequence number that could match a certain pathogen. Possible pathogens were determined according to the sequence number and other clinical tests.

### Interpretation of mNGS results

Given the lack of a standard method for interpreting mNGS results and the variety of reporting parameters among different sequencing platforms, we used the following criteria, which were derived and revised from prior literature on mNGS, to define clinically significant microbes (CSMs) (Miao et al., 2018; Xu et al., 2023). The threshold of sequenced reads in mNGS data analysis distinguishes true pathogens from background noise by enhancing specificity, reducing false positives caused by contamination or low-abundance commensals (Jia et al., 2021; Carbo et al., 2022; Du et al., 2022). Clinically, it ensures reliable pathogen identification, guiding targeted therapy while minimizing overdiagnosis.

1. Parasites: the reads number was ≥100 because of their large molecular weight and many broken fragments (Qin et al., 2021).
2. Other pathogens: the reads number was ≥3 (Miao et al., 2018; Xu et al., 2023).
3. For strictly pathogenic microorganisms such as *Mycobacterium tuberculosis* (MTB), Nocardia, Mycoplasma, and Aspergillus, the read number was ≥1 (Xu et al., 2023).
4. Intracellular bacteria such as *M. tuberculosis*, Legionella, and brucellosis: the read number was ≥1 because of their relatively low release into body fluids, leading to low detection sensitivity (Xu et al., 2023).
5. Some pathogenic microorganisms with thicker cell walls, such as fungi, the read number was ≥1 because nucleic acid extraction efficiency is low, resulting in a low clinical detection rate and sensitivity (Qin et al., 2021).

### Clinical composite diagnosis as the reference standard

The clinical composite diagnosis was determined by integrating clinical manifestations, laboratory tests, chest radiology, microbiological results (CMTs and mNGS), and treatment response, guided by the diagnostic criteria of CAP (Metlay et al., 2019) and HAP (Kalil et al., 2016). Two pulmonary infection specialists independently reviewed records of 296 patients (after excluding 21 with incomplete data from 317 enrolled). Etiology and pathogens were assessed, with disagreements resolved through discussion or consultation with a senior infectious disease expert. This yielded 224 pulmonary infections, 33 non-pulmonary infections, and 39 unexplained cases (**Figure 1**). Discordant mNGS and CMT results were reviewed by the expert panel to ensure clinical relevance, forming the reference standard for comparing pathogen detection rates.

### Statistical analysis

SPSS (version 26.0) was used to analyze the data. Continuous variables following a normal distribution were described as mean ± standard deviation, and the dependent t-test was used to compare between the groups. Continuous variables that did not follow a normal distribution were described as median (Q1, Q3), and the Wilcoxon rank test was used to compare between the groups. Categorical variables are described as n (%), and the chi-square test or Fisher’s exact test was used for categorical variables, as appropriate. Statistical significance was set at 5% (p < 0.05). GraphPad Prism (version 9.3) and Origin (version 9.9) were used to generate the graphs.

## Results

### Clinical characteristics

We compared and analyzed the sex, age, length of hospitalization, clinical manifestations, immune status, and comorbidities of the patients in the pulmonary infection group (PI) and the non-pulmonary infection group (NPI); the proportion of patients with fever (32.1% vs. 3%, p=0.001) was significantly higher in the PI group, and the proportion of patients with tumors (11.6% vs. 0, p=0.032) was significantly higher in the PI group (**Table 1**).

**Table 1:** Clinical characteristics of the patients.

Clinical characteristics	Total (257)	PI(224)	NPI(33)	P value
Sex(male)	**167 (65%)**	150 (67%)	17 (51.5%)	0.082
Age	55.49 ± 13.88	55.25 ± 14.26	57.15 ± 10.97	0.463
LOH(days)	12.72 ± 6.96	12.95 ± 7.2	11.18 ± 4.78	0.173
Clinical manifestation				
Fever	73 (28.4%)	72 (32.1%)	1 (3%)	**0.001**
Cough	178 (69.3%)	154 (68.8%)	24 (72.7%)	0.644
Expectoration	134 (52.1%)	116 (51.8%)	18 (54.4%)	0.767
Dyspnea	96 (37.4%)	80 (35.7%)	16 (48.5%)	0.157
Chest pain	53 (20.6%)	49 (21.9%)	4 (12.1%)	0.196
Chest distress	61 (23.7%)	53 (23.7%)	8 (24.2%)	0.942
Hemoptysis	37 (14%)	33 (14.7%)	3 (9.1%)	0.59
Immune deficiency	**35 (13.6%)**	33 (14.7%)	2 (6.1%)	0.275
Complication				
Hypertension	50 (19.5%)	43 (19.2%)	7(21.2%)	0.814
Cardiovascular disease	23 (8.9%)	20(8.9%)	3(9.1%)	1
Diabetes	36 (14%)	31(13.8%)	5(15.2%)	0.839
Malignant tumor	26 (10.1%)	26(11.6%)	0	**0.032**
Chronic pulmonary disease	69 (26.8%)	64 (28.4%)	5(15.2%)	0.104
Chronic renal disease	8 (3.1%)	8(3.6%)	0	0.601
Cerebrovascular disease	30 (11.7%)	27 (12.1%)	3 (9.1%)	0.777
Digestive diseases	8 (3.1%)	6 (2.7%)	2 (6.1%)	0.274

LOH, length of hospitalization.

The bold values indicate that the P-value is less than 0.05.

We also compared and analyzed the characteristics of pulmonary CT images and bronchoscopy and found that the proportional of bilateral lesions (63.4% vs. 81.8%, p=0.037), ground-glass opacity (21% vs. 45.5%, p=0.002), and interstitial lesions (17% VS 60.6%, p<0.001) was higher in the NPI group, whereas consolidation (29% vs. 9.1%, p=0.15) was higher in the PI group (**Table 2**). The bronchoscopy characteristics included normal, mucosal hyperemia, purulent and serous secretions, bronchial stenosis, and neoplasm, with no statistical difference between the two groups.

**Table 2:** Radiographic findings of the patients.

Radiographic finding and bronchoscopy	Total (257)	PI(224)	NPI(33)	P value
Radiographic finding				
Bilateral lesions	169 (65.8%)	142 (63.4%)	27 (81.8%)	**0.037**
Consolidation	68 (26.5%)	65 (29%)	3 (9.1%)	**0.015**
GGO	62 (24.1%)	47 (21%)	15 (45.5%)	**0.002**
Pleural effusion	50 (19.5%)	45 (20.1%)	5 (15.2%)	0.504
Patchy shadow	78 (30.4%)	69 (30.8%)	9 (27.3%)	0.68
Nodule	129 (50.2%)	114 (50.9%)	15 (45.5%)	0.56
Emptiness	28 (10.9%)	26 (11.6%)	2 (6.1%)	0.549
Interstitial lesion	58 (22.6%)	38 (17%)	20 (60.6%)	**0**
Bronchiectasis	49 (19.1%)	45 (20.1%)	4 (12.1%)	0.277
Mass shadow	35 (13.6%)	33 (14.7%)	2 (6.1%)	0.275
Bronchoscopy				
Normal	33 (12.8%)	30 (13.4%)	3(9.1%)	0.78
Mucosal hyperemia	132 (51.4%)	112 (50%)	20 (60.6%)	0.255
Secretion				0.076
Purulent secretion	40 (15.6%)	39(17.4%)	1 (3%)	
Serous secretion	31 (12.1%)	27 (12.1%)	4 (12.1%)	
Bronchial stenosis	22 (8.6%)	21(9.4%)	1 (3%)	0.224
Neoplasm	4 (1.6%)	3(1.3%)	1 (3%)	0.425

GGO, ground glass nodules.

The bold values indicate that the P-value is less than 0.05.

In the blood routine, compared to the NPI group, the PI group exhibited significantly lower levels of hemoglobin (Hb, 118.85 g/L vs. 129.64 g/L, p=0.006), red blood cell (RBC, 4.01 10^12/L vs 4.35 10^12/L, p=0.005), and hematocrit (Hct, 35.66 vs. 29.19, p=0.002); and higher levels of inflammatory markers, including C-reactive protein (CRP, 19.35 mg/L vs. 3.13 mg/L, p<0.001), erythrocyte sedimentation rate (ESR, 33 mm/60 min vs. 9.5 mm/60 min, p=0.004), ferritin (239.2 ng/mL vs. 134.5 ng/mL, p=0.009), and serum amyloid A (SAA, 31.9 mg/L vs. 4.1 mg/L, p=0.003). In the biochemical test, the PI group showed a significant increase in γ-glutamyl transpeptidase (γGT, 28 U/L vs. 21 U/L, p=0.013) and a decrease in serum albumin (ALB, 36.6 g/L vs. 39.5 g/L, p=0.01). Regarding coagulation function, the PI had significantly elevated fibrinogen levels (FIB, 4.76 g/L vs. 3.6 g/L, p<0.001) (**Table 3**).

**Table 3:** Laboratory findings of the patients.

Laboratory findings	Total (257)	PI(224)	NPI(33)	P value
WBC(G/L)	6.37 (4.97, 8.49)	6.5 (5.02, 8.69)	6.04 (4.85, 7.17)	0.105
RBC(T/L)	4.05 ± 0.69	4.01 ± 0.69	4.35 ± 0.55	**0.006**
Hb(g/L)	120.25 ± 20.5	118.85 ± 20.42	129.64 ± 18.8	**0.005**
Hct(%)	36.12 ± 6.12	35.66 ± 6.18	39.19 ± 4.67	**0.002**
PLT(G/L)	254.22 ± 102.21	257.75 ± 103.99	230.45 ± 87.07	0.153
Neutrophils(%)	67.92 ± 12.97	68.48 ± 13.16	64.16 ± 10.99	0.074
Lymphocyte(%)	21.38 ± 10.97	20.89 ± 11.06	24.63 ± 9.85	0.067
CRP(mg/L)	14.5 (3.14, 52.18)	19.35 (3.64, 59.8)	3.13 (3.11, 5.11)	**0**
PCT(ng/ml)	0.13 (0.13, 0.15)	0.13 (0.13, 0.15)	0.13 (0.13, 0.13)	0.36
ESR(mm/h)	27 (8, 63.5)	33 (10, 66)	9.5 (5.75, 31.75)	**0.004**
Fer(ug/L)	218.5 (93.4, 428.2)	239.2 (98.25, 442.4)	134.5 (54.7, 175.4)	**0.009**
SAA(mg/L)	16.35 (4.4, 194.15)	31.9 (4.9, 278.7)	4.1 (2.1, 17)	**0.003**
TB(umol/L)	10.96 ± 5.3	10.95 ± 5.49	11.04 ± 3.9	0.929
DB(umol/L)	3.81 ± 2.26	3.86 ± 2.35	3.46 ± 1.42	0.342
ALT(U/L)	26.72 ± 25.9	27.46 ± 27.1	21.82 ± 15.25	0.244
AST(U/L)	21 (17, 28)	21 (17, 28)	22 (17, 27.5)	0.944
ALP(U/L)	75 (63, 95.75)	75 (61, 98)	74 (68, 87.5)	0.849
γGT(U/L)	26 (15, 45)	28 (15, 49)	21 (14, 27)	**0.013**
TP(g/L)	63.52 ± 7.45	63.38 ± 7.64	64.41 ± 6.05	0.461
ALB(g/L)	37.2 (32.4, 40.78)	36.6 (31.8, 40.5)	39.5 (37.2, 41.8)	**0.01**
Cr(umol/L)	76.04 ± 83.42	77.22 ± 88.92	68.25 ± 25.53	0.566
BUN(mmol/L)	5.52 ± 3.9	5.58 ± 4.11	5.14 ± 1.94	0.543
UA(umol/L)	290.71 ± 101.55	287.37 ± 98.41	312.87 ± 119.75	0.179
Cystatin C(mg/L)	1.06 ± 1.03	1.09 ± 1.1	0.92 ± 0.24	0.488
BG(mmol/L)	5.41 ± 1.6	5.4 ± 1.52	5.46 ± 2.02	0.865
CK(U/L)	69.87 ± 78.17	67.46 ± 81.33	86.43 ± 49.62	0.278
LDH(U/L)	250.28 ± 129.86	250.46 ± 134.48	249 ± 92.55	0.96
D-dimer(mg/L)	0.68 (0.32, 1.86)	0.72 (0.32, 1.96)	0.42 (0.29, 0.92)	0.054
FIB(g/L)	4.51 (3.37, 6.09)	4.76 (3.48, 6.18)	3.6 (2.99, 4.23)	**0**
PT(s)	13.49 ± 1.05	13.54 ± 1.07	13.19 ± 0.77	0.09
APTT(s)	39.16 ± 5.04	39.37 ± 5.15	37.61 ± 3.85	0.072

WBC, white blood cell count; RBC, red blood cell count; Hb, hemoglobin; Hct, hematocrit; PLT, platelet count; CRP, C-reactive protein; PCT, procalcitonin; ESR, erythrocyte sedimentation rate; Fer, ferritin; SAA, serum amyloid A; TB, total bilirubin; DB, direct bilirubin; ALT, alanine aminotransferase; AST, aspartate aminotransferase; ALP, alkaline phosphatase; γGT, γ -glutamyl transpeptidase; TP, total protein; ALB, albumin; Cr, creatinine; BUN, blood urea nitrogen; UA, uric acid;BG, blood glucose; CK, creatine kinase; LDH, lactate dehydrogenase; FIB, fibrinogen; PT, prothrombin time; APTT, activated partial thromboplastin time.

The bold values indicate that the P-value is less than 0.05.

### Pathogen profiles

**Figure 2:**
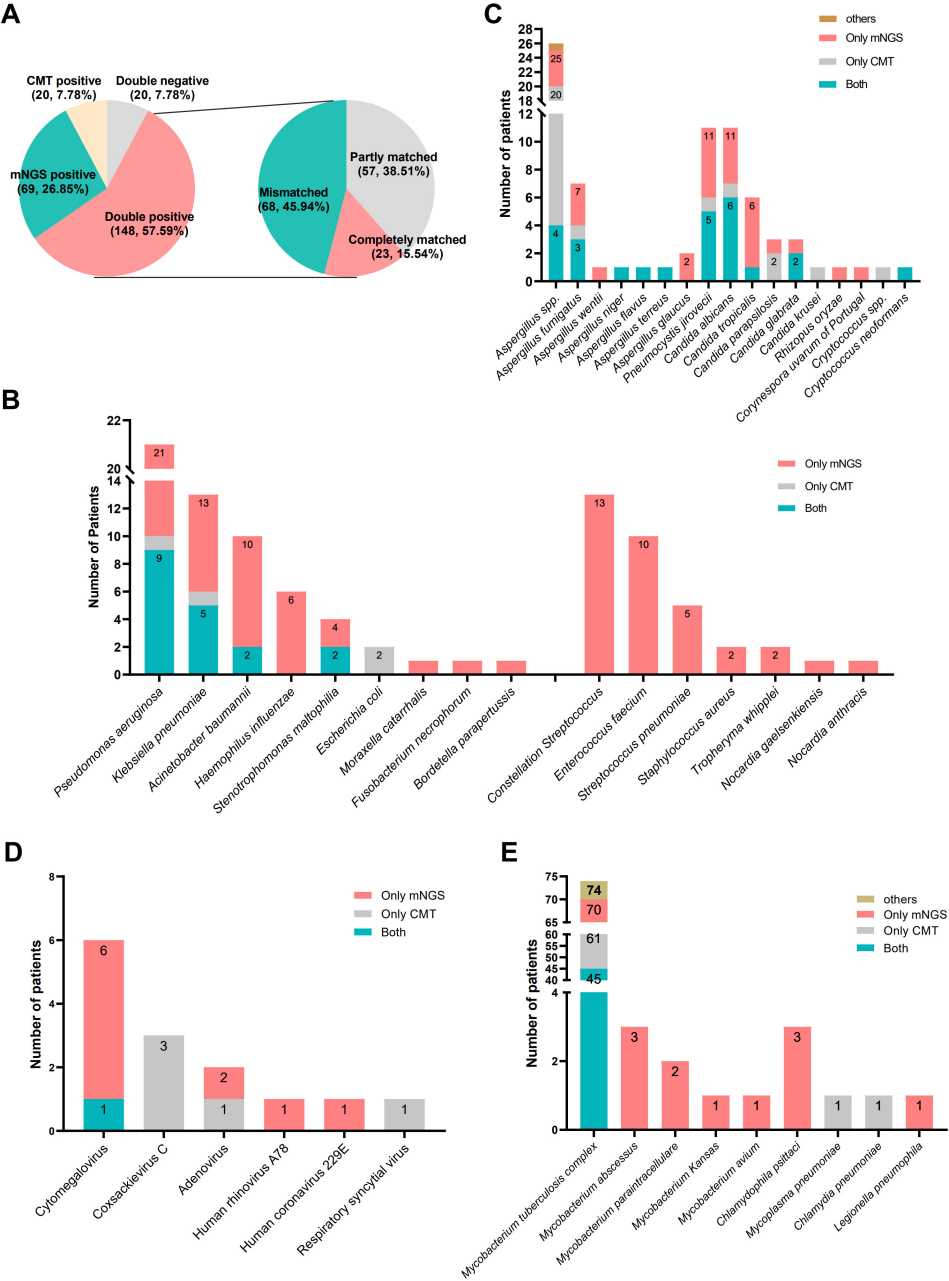
Pathogen profiles. **(A)** The concordance analysis of CMT and mNGS; **(B)** types and number of fungi detected by CMT and mNGS; **(C)** types and number of bacteria detected by CMT and mNGS; **(D)** types and number of virus detected by CMT and mNGS; **(E)** types and number of atypical pathogens detected by CMT and mNGS.

Using the final clinical diagnosis as the reference standard, mNGS achieved a sensitivity of 87.95% (95% CI: 86.83%–94.46%) and a specificity of 39.39% (95% CI: 22.91%–57.86%), while CMT demonstrated a sensitivity of 69.64% (95% CI: 63.17%–75.59%) and a specificity of 63.64% (95% CI: 45.12%–79.60%) (**Table 4**). Concordance analysis of CMT and mNGS results showed that 148 patients (57.59%) were double-positive, 20 (7.78%) were double-negative, 69 (26.85%) were positive for mNGS but negative for CMT, and 20 (7.78%) were positive for CMT but negative for mNGS (**Figure 2A**). Among 148 double-positive patients, 23 (15.54%) were completely matched between mNGS and CMT, 57 (38.51%) were partially matched, and 68 (45.94%) were mismatched.

**Table 4:** Comparison of diagnostic performance between mNGS and CMTs in patients with suspected pneumonia.

Methods	Results	Clinical diagnosis positive	Clinical diagnosis negative	Accuracy	Sensitivity	Specificity
mNGS	Positive	197	20	81.71% (76.43%–86.24%)	87.95% (86.83%–94.46%)	39.39% (22.91%–57.86%)
	Negative	27	13			
CMT	Positive	156	12	68.87% (62.82%–74.48%)	69.64% (63.17%–75.59%)	63.64% (45.12%–79.60%)
	Negative	68	21			

As shown in **Figures 2C~E**, we counted the number of pathogens detected by CMT and mNGS and showed the number of detected fungi, bacteria, viruses, and atypical pathogens in the form of bar charts, respectively. The most frequently identified pathogens were *Mycobacterium tuberculosis*, *Aspergillus* spp., *Pneumocystis jiroveci*, *Candida albicans*, *Pseudomonas aeruginosa*, *Klebsiella pneumonia*, and *Acinetobacter baumannii*. Multiple species, including *Nocardia* spp., *Streptococcus constellatus*, *Enterococcus faecalis*, *Streptococcus pneumoniae*, *Haemophilus influenzae*, non-tuberculous Mycobacterium tuberculosis, *Chlamydia psittaci*, *Legionella pneumophila*, were detected solely by mNGS.

### Comparison of positive rates among different groups

**Figure 3:**
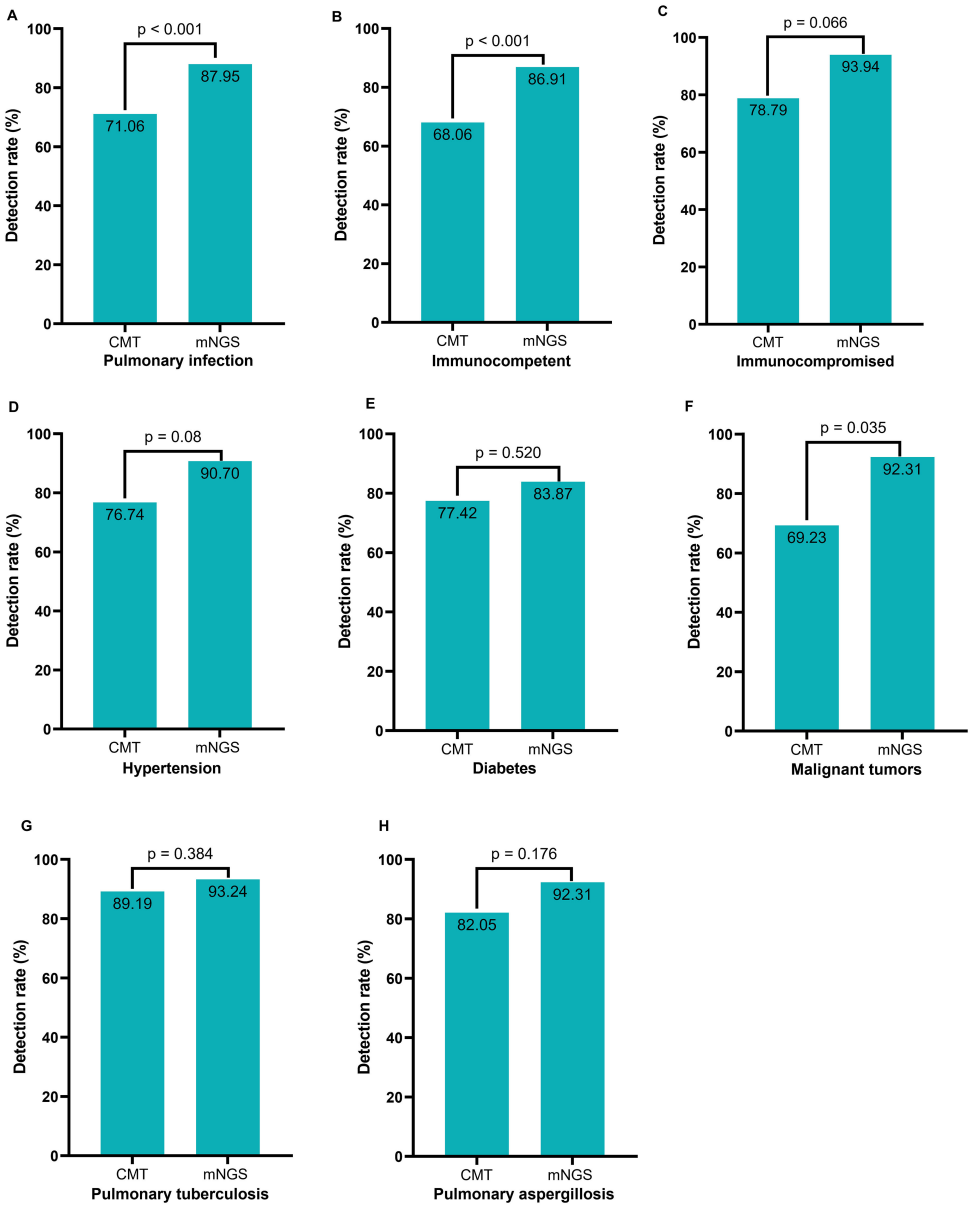
Comparison of positive rates among various subgroups between CMT and mNGS. **(A)** All pulmonary infection patients; **(B)** Pulmonary infection patients with normal immune function; **(C)** Pulmonary infection patients with compromised immune function; **(D)** Pulmonary infection patients with hypertension; **(E)** Pulmonary infection patients with diabetes; **(F)** Pulmonary infection patients with tumor; **(G)** Pulmonary tuberculosis patients; **(H)** Pulmonary aspergillosis patients.

We categorized patients with pulmonary infection (PI groups) (**Figure 3A**) into various subgroups based on their immune status and comorbidities. The subgroups included immunocompromised patients (**Figure 3B**), immunocompetent patients (**Figure 3C**), patients with hypertension (**Figure 3D**), patients with diabetes (**Figure 3E**), patients with malignant tumors (**Figure 3F**), patients diagnosed with pulmonary tuberculosis (**Figure 3G**), and patients diagnosed with pulmonary aspergillosis (**Figure 3H**). We also compared the detection positive rates between CMT and mNGS in various subgroups, which showed that the overall positive rate of mNGS was significantly higher in patients with pulmonary infections (87.95% vs. 71.06%, p<0.001). The positive rate of mNGS was significantly higher in immunocompetent patients (86.91% vs. 68.08%, p<0.001) and malignant tumors (92.31% vs. 69.23%, p=0.035). The detection rate was not significantly different between CMT and mNGS in immunocompromised patients (93.94% vs. 78.79%, p=0.066), in patients with hypertension (90.70% vs. 76.74%, p=0.08), in patients with diabetes (83.87% vs. 77.42%, p=0.52), pulmonary tuberculosis (93.24% vs. 89.19%, p=0.384), or pulmonary aspergillosis (92.31% vs. 82.05%, p=0.176).

### The impact of mNGS on the diagnosis and treatment

The pathogens reported by the mNGS results may not always be the causative agents. Therefore, the positive rate of mNGS cannot be used as an objective measure to assess its impact on the diagnosis and treatment of patients with suspected lung infections. To further explore the impact of mNGS results on diagnosis and treatment in actual clinical practice, we carefully categorized the various possible impacts; the detailed classifications are listed in **Supplementary Table 1** and **Supplementary Table 2**. For pathogen diagnosis, mNGS has three clinical impacts: positive impact, non-impact, and negative impact. The positive impact can be categorized into three categories: D1, mNGS result was quicker than CMT; D2, co-infection was diagnosed based on mNGS; and D3, mNGS result contributed to pathogen identification. The non-impact also had three categories: D4, mNGS results were negative; D5, mNGS detected the same pathogens as CMT and did not detect them earlier than CMT; and D6, the microbes detected by mNGS were assessed as unlikely pathogens. The negative impact had only one category: D7, Lung infection pathogens were undetected by mNGS and without suspected pathogen detection. Regarding pathogen treatment, mNGS had only two clinical impacts: positive impact and non-impact. The positive impact was divided into four categories: T1, initiation of appropriate antibiotic treatment; T2, guidance of antimicrobial escalation; T3, guidance of antimicrobial de-escalation; and T4, confirmation of empiric treatment. The non-impact had two categories: T5, mNGS results were positive but treatment was not adjusted; T6, mNGS results was negative but treatment was not adjusted.

**Figure 4:**
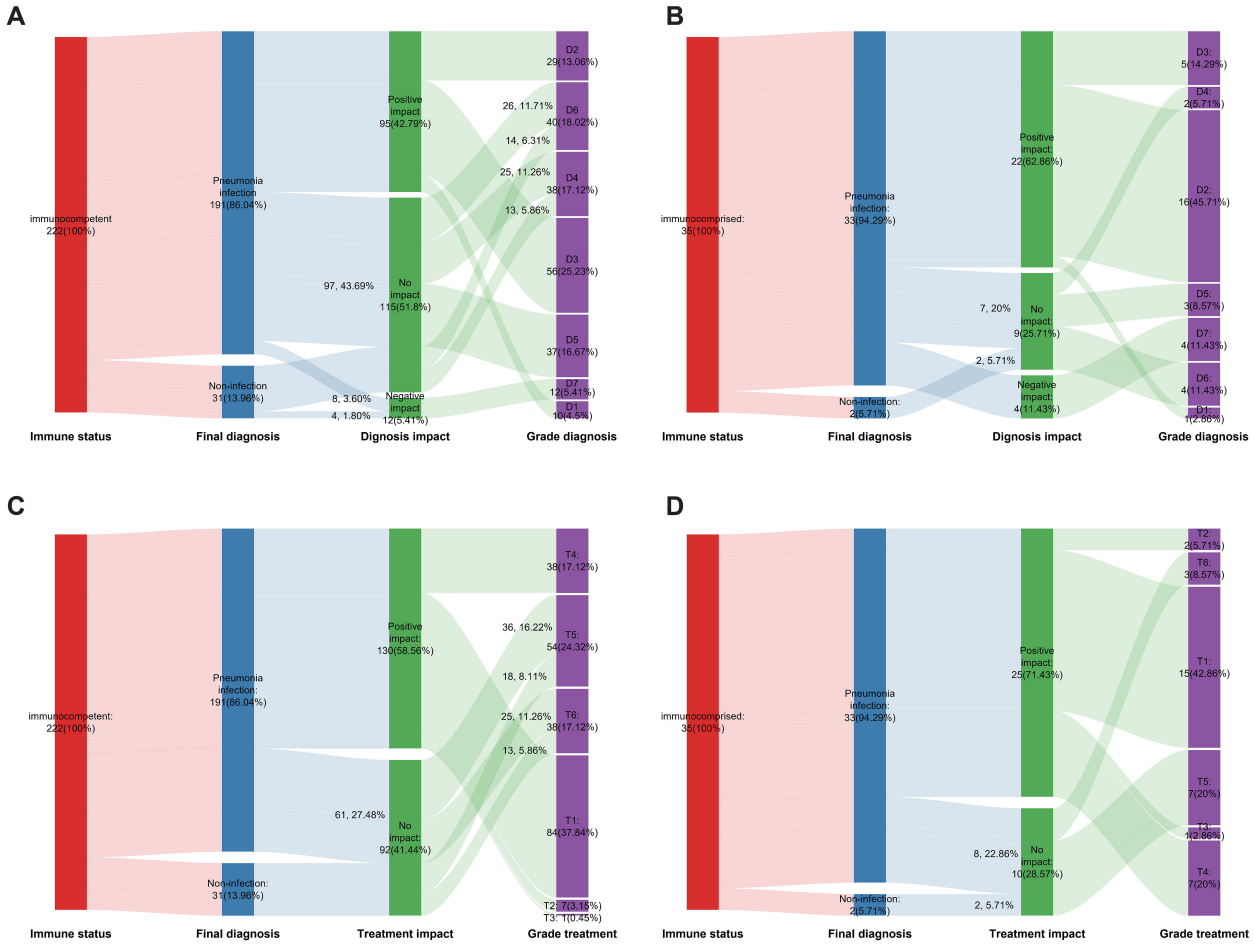
The impact of mNGS on the diagnosis and treatment. **(A)** The effect of mNGS on pathogen diagnosis among immunocompetent patients; **(B)** The effect of mNGS on pathogen diagnosis among immunocompromised patients; **(C)** The effect of mNGS on treatment among immunocompetent patients; **(D)** The effect of mNGS on treatment among immunocompromised patients.

A total of 222 immunocompetent patients were suspected to have a pulmonary infection. The mNGS assay had a positive impact on the final diagnosis of the causative agent in 95 patients, representing 42.79% of immunocompetent patients. Among these patients, D1, D2, and D3 accounted for 4.5%, 13.06%, and 25.23%, respectively. The mNGS assay did not have an impact on the diagnosis in 115 patients, in which D4, D5, and D6 had proportions of 17.12%, 16.67%, and 18.02%, respectively. It had a negative impact on the diagnosis in 12 patients (D7, 5.41%) (**Figure 4A**). The mNGS assay had a positive impact on pathogen treatment in 130 (58.56%) immunocompetent patients but had no impact in 92 (41.44%) patients. Among patients with a positive impact, T1 accounted for the largest proportion (37.84%), whereas T5 had the highest proportion of patients with no impact (24.32%) (**Figure 4C**). In our study, there were 35 immunocompromised patients, in which the mNGS assay had a positive impact, non-impact, and negative impact on the final diagnosis in 22 patients (62.86%), 9 patients (25.71%), and 4 patients (11.43%), respectively. The positive impact was mainly categorized as D2 (45.71%) and the non-impact as D6 (11.43%) (**Figure 4B**). The positive impact and non-impact of the mNGS assay on the treatment of immunocompromised patients were observed in 25 and 10 patients, respectively (**Figure 4D**).

To thoroughly compare the impact of mNGS results on diagnosis and treatment outcomes between immunocompromised and immunocompetent patients, we combined the categories of negative impact and non-impact to serve as the control group for positive impact. Chi-square tests were performed to evaluate differences. Compared to immunocompetent patients, mNGS in immunocompromised individuals with suspected lung infections demonstrated significantly higher rates of accurate diagnosis (62.86% vs. 42.79%, p = 0.027) and a trend toward more effective treatment outcomes (71.43% vs. 58.56%, p = 0.148), though the latter did not reach statistical significance.

## Discussion

Our study revealed a significantly higher prevalence of fever (32.1% vs. 3%, p=0.001) among patients diagnosed with pulmonary infections than among those without pulmonary infections. Patients with malignant tumors (11.6% vs. 0, p=0.032) were more prone to developing pulmonary infections than those with hypertension, cardiovascular disease, and diabetes. However, in this study, the number of patients with pulmonary infections complicated by malignant tumors is relatively small (n=26). Therefore, this finding is considered as exploratory, and further validation with larger clinical samples is needed to confirm the correlation between pulmonary infections and tumors. The likelihood of infection is significantly elevated in the presence of fever and abnormal lung infiltration. A comparative analysis of pulmonary CT images between the PI and NPI groups showed that consolidation (29% vs. 9.1%, p=0.15) was more prevalent in patients with lung infections. In contrast, bilateral involvement (63.4% VS 81.8%, p<0.001), ground-glass opacity (21% vs. 45.5%, p=0.002), and interstitial lesions (17% VS 60.6%, p<0.001) were more common in non-pulmonary infections. The comparative analysis of blood routine, biochemical and other blood test results indicated that patients in the lung infection group exhibited lower levels of Hb (118.85 g/L vs. 129.64 g/L, p=0.006) and serum albumin (36.6 g/L vs. 39.5 g/L, p=0.01), as well as higher levels of inflammatory markers, including CRP (19.35 mg/L vs. 3.13 mg/L, p<0.001), ESR (33 mm/60 min vs. 9.5 mm/60 min, p=0.004), ferritin (239.2 ng/mL vs. 134.5 ng/mL, p=0.009), SAA (31.9 mg/L vs. 4.1 mg/L, p=0.003), and FIB (4.76 g/L vs. 3.6 g/L, p<0.001). These findings demonstrate that, in our clinical practice, patients who exhibited fever, had malignant tumors and abnormalities such as consolidation on CT images, and showed elevated inflammatory markers on blood tests were more likely to have lung infections. These findings are consistent with observations in clinical practice and have been documented in published studies (Akinosoglou et al., 2013; Aliberti et al., 2021; Musher and Thorner, 2014; Valvani et al., 2019). It may also have a certain guiding significance for selecting appropriate patients with suspected pulmonary infections to perform BALF mNGS in the future.

Several published studies have shown that the positive rate and sensitivity of mNGS are superior to those of CMT, especially in the diagnosis of mixed infections, critically ill patients, and immunocompromised individuals (Deng et al., 2022; Parize et al., 2017; Zhong et al., 2023). A prospective multicenter study demonstrated that untargeted next-generation sequencing detected significantly more clinically relevant viruses and bacteria than conventional methods (36% vs 11%, p<0.001) in immunocompromised adults, with high negative predictive value (64/65, 95% CI 0.95-1), highlighting its diagnostic potential (Parize et al., 2017). Deng et al. (2022) compared the diagnostic performance of bronchoalveolar lavage fluid (BALF) mNGS versus CMT in pediatric pneumonia. Their results showed that mNGS had significantly higher overall pathogen detection rates (91.3% vs. 59.2%, p<0.001), with superior sensitivity for bacterial and viral infections. However, mNGS exhibited lower sensitivity for *Mycoplasma pneumoniae* (42.1% vs. 100%) compared to CMT. Additionally, mNGS identified virus-bacteria co-infections as the most prevalent polymicrobial pattern. Zhong et al. (2023) found that mNGS showed significantly higher detection rates than conventional culture (93.3% vs. 29.3%, p<0.0001) in AIDS patients with pulmonary infections. mNGS also identified polymicrobial infections in 60% of these patients, further emphasizing its superior diagnostic value. Nevertheless, Peng et al. (2021) retrospectively analyzed 60 critically ill immunocompromised patients with suspected pneumonia and compared the diagnostic performance of BALF mNGS and CMTs. Their study found that the overall diagnostic accuracy was similar between mNGS and CMTs. While mNGS was better at identifying viral pneumonia, it was less accurate for fungal infections. The authors suggested that combining mNGS and CMTs may be the optimal diagnostic strategy.

Collectively, the diagnostic effectiveness of mNGS varies depending on the specific types of pathogens, types of specimens being tested, and patient characteristics. Regardless of cost, the combination of mNGS and CMT remains the most effective diagnostic method (Qu et al., 2022).

For certain pathogens that lack rapid and effective diagnostic methods in some medical institutions, such as *C. psittaci*, *P. jirovecii*, NTM, *L. pneumophila*, mNGS detection methods also have unique benefits (Chen et al., 2023; Du et al., 2023; Huang et al., 2023; Tang et al., 2023), which is consistent with our findings (**Figure 2**). This study showed that mNGS can detect a wider range of pathogens compared to CMT. It was able to identify pathogens that typically require advanced culture conditions, such as *S. pneumoniae*, *H. influenzae*, *S. constellatus*, *Nocardia* spp.

Although both mNGS and CMT showed a high positive rate in patients with suspected lung infections, mNGS showed a significantly higher rate (**Figure 3A** **).** There was also a relatively high level of disagreement between the two methods (**Figure 2A**), which could be due to the broad range of tests included in the CMT, such as the T-SPOT, sputum culture, BALF GM test, and blood G test. CMT positivity cannot completely confirm the corresponding pathogen infection but will be included in the positive statistical results.

This study found comparable detection rates for *M. tuberculosis* and Aspergillus between mNGS and CMT (**Figures 3G, H**). CMT, including sputum smear, BALF X-pert, T-SPOT, and galactomannan tests, is cost-effective for routine cases. But mNGS excels in complex scenarios, detecting *M. tuberculosis* in atypical or CMT-negative cases and identifying Aspergillus species for targeted therapy. mNGS also detects co-infections, enhancing diagnostic scope. While CMT is preferred for routine tuberculosis screening, mNGS may be more economical when invasive procedures are needed. mNGS is not recommended for routine use but adds value in challenging cases. However, subgroup analyses are often underpowered (**Supplementary Table 3**), with most sample sizes below those required for 80% power, except for immunocompetent and pulmonary infection subgroups. Thus, subgroup findings are exploratory, requiring larger studies for validation. Unlike previous studies, we conducted an analysis to assess the impact of the mNGS test on the final diagnosis and adjustment of the treatment regimen for each patient to gain a deeper understanding of its impact on actual clinical diagnosis and therapy. The mNGS diagnosis impact (D1–D7) and treatment impact (T1–T6) classifications were enhanced by evaluating inter-rater reliability, yielding Cohen’s kappa values of 0.866 (95% CI: 0.796–0.937) for diagnosis (**Supplementary Table 4**) and 0.868 (95% CI: 0.783–0.953) for treatment (**Supplementary Table 5**), indicating strong agreement. Our results show that BALF mNGS has several beneficial effects on diagnosis and treatment. Specifically, mNGS assists in the diagnosis of mixed infections and identification of infectious pathogens. Moreover, it aided in initiating appropriate antibiotic treatment and confirmed the effectiveness of empirical treatment. Further subgroup analysis showed that BALF mNGS in immunocompromised patients suspected of having pulmonary infection could have more positive effects on diagnosis (42.79% vs. 62.86%, p= 0.0267) and treatment (58.56% vs. 71.43%, p= 0.148).

Liang and colleagues (Liang et al., 2022) conducted a study to analyze the effect of BALF mNGS on the treatment of patients with suspected lower respiratory tract infections. The results showed that 3.6% of patients were degraded by antibiotics (reducing the antibacterial spectrum or reducing the types of antibiotics), 23.6% were upgraded (increasing the types of antibiotics or changing to broad-spectrum antibiotics), 60.7% remained unchanged, and 12.1% were transferred to a lung hospital. Studies have shown that the majority of patients do not adjust their treatment plan following mNGS testing; however, the mNGS results verify the accuracy of empirical treatment, which is consistent with our research results (**Figure 4**). We are confident that the results of mNGS will have a positive impact on this particular group of patients. Xiao and colleagues (Jin et al., 2022) also found that the ultimate diagnosis and treatment adjustment of cases primarily relied on the combined findings of mNGS, CT, other tests, and clinical features. Modifications and adjustments were made solely based on the results of mNGS in 32 (32/246, 13.01%) and 23 (23/246, 9.35%) cases, respectively. These modifications have beneficial effects on the disease progression and prognosis of these patients. Most of these patients were infected with *M. tuberculosis*, NTM, or atypical pathogens.

Han and his colleagues (Han et al., 2023) discovered that the results of plasma mNGS had a positive effect on 83 patients (57.1%). This was achieved by accurately diagnosing or excluding infections and initiating targeted therapies. However, only 32.4% (11/34) of the negative mNGS tests showed a positive impact, suggesting that plasma mNGS testing alone may not be a powerful tool for ruling out infections in clinical settings.

Although the tested samples and classification criteria for the impact on diagnosis and treatment may vary, our study classified a negative result from the NGS test as having no effect on the clinical diagnosis. However, our results showed that for suspected pneumonia patients with normal immunity, approximately half of the mNGS results had a positive impact on the diagnosis and treatment. More clinical trials and studies are required to gain a deeper understanding of the appropriate individuals to test, the optimal timing for testing, and how to optimize the diagnostic efficiency and socioeconomic benefits of mNGS for pulmonary infectious diseases. Furthermore, this manual classification system prioritizes diagnostic context over automated interpretation. Retrospective design allows granular analysis but limits real-time clinical generalizability. Current framework requires specialized expertise, making it less suitable for emergency settings where rapid decision-making predominates.

In clinical practice, we frequently encounter patients presenting with either radiological pulmonary abnormalities and/or respiratory symptoms, which necessitates comprehensive clinical evaluation to differentiate true pulmonary infections from other pathologies and decide on antimicrobial therapy initiation. Our study specifically addressed this diagnostic challenge through rigorous case selection criteria (detailed in “Study design and patient population”), where the cohort potentially included non-infectious pulmonary conditions such as interstitial lung disease, pulmonary edema, and neoplastic lesions. Critical analysis revealed that patients demonstrating specific clinical features (e.g., febrile presentation), comorbid conditions (particularly malignancies), characteristic CT findings (consolidation or ground-glass opacities), and laboratory abnormalities (elevated inflammatory markers) showed significantly higher probabilities of confirmed pulmonary infections. These findings systematically validate our clinical experience through three key dimensions: 1. Diagnostic Triangulation: Integration of microbiological, radiological, and laboratory evidence. 2. Risk Stratification: Identification of high-yield clinical predictors for infection likelihood. 3. Diagnostic Stewardship: Guidance for judicious antimicrobial use in ambiguous cases. This evidence-based alignment between empirical clinical judgment and systematic research outcomes reinforces the necessity of multidimensional assessment in pulmonary infection diagnosis. The concordance observed provides scientific validation for current diagnostic protocols.

Compared to CMTs, mNGS has a higher initial cost. This is due to the expensive sequencer, sophisticated reagents, and significant computational resources required for data analysis. As a result, the cost of a single mNGS test is often several times higher than that of traditional methods. However, mNGS provides a comprehensive view of the microbial community in a sample within a relatively short time, typically within 24–48 hours. This rapid turnaround time can potentially reduce the overall cost of care by enabling more targeted and timely treatment, thereby avoiding prolonged and inappropriate empirical therapy (Miller and Chiu, 2021). In contrast, CMTs have limitations in detecting fastidious or unculturable pathogens. For example, many viruses and some intracellular bacteria are difficult to identify using conventional culture methods, which can lead to misdiagnosis or delayed diagnosis. This, in turn, affects the effectiveness of treatment and patient outcomes. In contrast, mNGS can detect a wide range of pathogens, including novel and emerging ones, without prior knowledge of the causative agent. This enhanced diagnostic capability can lead to more effective treatment strategies and improved patient prognosis. In summary, while the initial costs of implementing NGS technologies in infectious disease diagnostics are high, the potential for improved diagnostic accuracy, reduced hospital stays, and more effective patient management can justify these expenses. The continued development and refinement of NGS technologies, alongside advances in bioinformatics and data analysis, are expected to further enhance their cost-effectiveness and clinical utility in the future (Liu and Ma, 2024; Haslam, 2021).

This study has several limitations. First, most patients underwent only mNGS DNA testing, potentially missing RNA viruses. Second, as a single-center study with few immunocompromised patients, generalizability is limited. Third, the retrospective design introduced selection bias, as mNGS was often used for complex cases or patients with better socioeconomic status, restricting applicability to broader populations. Fourth, missing data in routine tests or CMTs, particularly in milder cases, may have affected precision. A complete case analysis (**Supplementary Table 6**), including only patients with complete data, confirmed robust results. Fifth, the lack of standardized mNGS interpretation criteria and unblinded diagnostic adjudication may have introduced bias, though mitigated by expert consensus and predefined thresholds. Finally, cost-effectiveness was not evaluated. Future prospective, multicenter studies with larger cohorts, standardized criteria, blinded adjudication, and cost analyses are needed to validate our findings.

## Data Availability

The raw sequence data reported in this paper have been deposited in the Genome Sequence Archive (Genomics, Proteomics & Bioinformatics 2021) in National Genomics Data Center (Nucleic Acids Res 2022), China National Center for Bioinformation / Beijing Institute of Genomics, Chinese Academy of Sciences (GSA: CRA026168) that are publicly accessible at https://ngdc.cncb.ac.cn/gsa.
