# Supplementary material for: The impact of bronchoalveolar lavage fluid metagenomics next-generation sequencing on the diagnosis and management of patients with suspected pulmonary infection
Source: Front Cell Infect Microbiol. 2025 Jun 23;15:1521641. doi: 10.3389/fcimb.2025.1521641 (PMC12230576; doi:10.3389/fcimb.2025.1521641)
Supplement: Supplementary file 1 [file Table1.docx]

**Supplement table 1. The clinical impact of BALF mNGS on diagnosis.**

| **clinical impact** | **hierarchy** | **descriptive** |
| --- | --- | --- |
| Positive | D1 | mNGS result was quicker than CMT |
|  | D2 | Co-infection was diagnosed based on mNGS |
|  | D3 | mNGS result contributed to pathogen identification |
| No impact | D4 | mNGS results were negative |
|  | D5 | mNGS detected the same pathogens as CMT and did not detect them earlier than CMT |
|  | D6 | The microbes detected by mNGS were assessed as unlikely pathogens |
| Negative | D7 | Lung infection pathogens were undetected by mNGS and without suspected pathogen detection |
