# Supplementary material for: The impact of bronchoalveolar lavage fluid metagenomics next-generation sequencing on the diagnosis and management of patients with suspected pulmonary infection
Source: Front Cell Infect Microbiol. 2025 Jun 23;15:1521641. doi: 10.3389/fcimb.2025.1521641 (PMC12230576; doi:10.3389/fcimb.2025.1521641)
Supplement: Supplementary file 2 [file Table2.docx]

**Supplement table 2. The clinical impact of BALF mNGS on streatment.**

| **clinical impact** | **hierarchy** | **descriptive** |
| --- | --- | --- |
| Positive | T1 | Initiation of the appropriate antibiotic treatment |
|  | T2 | Antibiotics escalation |
|  | T3 | Antibiotics de-escalation |
|  | T4 | Confirmed empirical treatments |
| No impact | T5 | mNGS results was positive, but treatment was not adjusted |
|  | T6 | mNGS results was negative, but treatment was not adjusted |
