# Supplementary material for: The impact of bronchoalveolar lavage fluid metagenomics next-generation sequencing on the diagnosis and management of patients with suspected pulmonary infection
Source: Front Cell Infect Microbiol. 2025 Jun 23;15:1521641. doi: 10.3389/fcimb.2025.1521641 (PMC12230576; doi:10.3389/fcimb.2025.1521641)
Supplement: Supplementary file 3 [file Table3.docx]

**Supplementary Table 3. Power calculations for the subgroups analysis.**

| Subgroup | Sample Size (N) | mNGS Positive Rate (%) | CMT Positive Rate (%) | Effect Size (h) | Power (%) | Required Sample Size (80% Power) |
| --- | --- | --- | --- | --- | --- | --- |
| Pulmonary Infection | 224 | 87.95 | 71.06 | 0.427 | 99.48 | 87 |
| Immunocompromised | 33 | 87.95 | 71.06 | 0.427 | 41.09 | 87 |
| Immunocompetent | 191 | 86.91 | 68.08 | 0.460 | 99.44 | 75 |
| Hypertention | 43 | 90.70 | 83.87 | 0.207 | 16.01 | 368 |
| Diabetes | 31 | 83.87 | 77.42 | 0.164 | 9.88 | 586 |
| Malignant Tumors | 26 | 92.31 | 69.23 | 0.614 | 60.03 | 421 |
| Pulmonary tuberculosis | 75 | 93.33 | 89.33 | 0.143 | 14.15 | 767 |
| Pulmonary aspergillosis | 39 | 92.31 | 82.05 | 0.313 | 28.21 | 161 |

Notes: Power calculations were performed using the pwr.2p.test function in R package pwr, assuming a significance level of α=0.05.
