# Supplementary material for: The impact of bronchoalveolar lavage fluid metagenomics next-generation sequencing on the diagnosis and management of patients with suspected pulmonary infection
Source: Front Cell Infect Microbiol. 2025 Jun 23;15:1521641. doi: 10.3389/fcimb.2025.1521641 (PMC12230576; doi:10.3389/fcimb.2025.1521641)
Supplement: Supplementary file 4 [file Table4.docx]

**Supplementary Table 4. Inter-Rater Reliability for mNGS Diagnosis Impact Classification**

| Parameter | Value |
| --- | --- |
| Number of Patients Assessed (Total) | 296 |
| Number of Patients Reviewed by Third Expert | 100 |
| Cohen’s Kappa Coefficient | 0.866 |
| 95% Confidence Interval | 0.796–0.937 |
| Standard error | 0.03598 |

Note: The mNGS impact was classified into seven categories (D1–D7), ranging from no impact (D1) to critical diagnostic contribution (D7). A random subset of 100 patients was blindly assessed by a third specialist to evaluate inter-rater reliability.
