# Supplementary material for: The impact of bronchoalveolar lavage fluid metagenomics next-generation sequencing on the diagnosis and management of patients with suspected pulmonary infection
Source: Front Cell Infect Microbiol. 2025 Jun 23;15:1521641. doi: 10.3389/fcimb.2025.1521641 (PMC12230576; doi:10.3389/fcimb.2025.1521641)
Supplement: Supplementary file 6 [file Table6.docx]

**Supplementary Table 6. Sensitivity analysis**

|  | Analysis type | |
| --- | --- | --- |
|  | Primary Analysis (Median imputation) | Complete case analysis |
| mNGS sensitivity (95% CI) | 87.95% (86.83%–94.46%) | 81.11% (71.49%–88.59%) |
| mNGS specificity (95% CI) | 39.39% (22.91%–57.86%) | 39.13% (19.71%–61.46%) |
| CMT sensitivity (95% CI) | 69.64% (63.17%–75.59%) | 71.11% (60.60%–80.18%) |
| CMT specificity (95% CI) | 63.64% (45.12%–79.60%) | 60.87% (38.54%–80.29%) |
| Immunocompromised patients (mNGS vs CMT positive, P value) | 93.94% vs. 78.79%, p=0.066 | 93.75% vs. 93.75%, p=1 |
| Immunocomptent patients (mNGS vs CMT positive, P value) | 86.91% vs. 68.08%, p<0.001 | 74.23% vs. 59.79%, p=0.0329 |
| Diagnosis impact (immunocompromised vs. immunocompetent, p value) | 62.86% vs. 42.79%, p = 0.0267 | 56.25% vs. 39.18%, p=0.2013 |
| Treatment impact (immunocompromised vs. immunocompetent, p value) | 71.43% vs. 58.56%, p = 0.1481 | 68.75% vs. 47.42%, p=0.1155 |

Note: The complete case analysis included 113 patients with no missing data.
